# Integrative Phylogenetics: Tools for Palaeontologists to Explore the Tree of Life

**DOI:** 10.3390/biology11081185

**Published:** 2022-08-07

**Authors:** Raquel López-Antoñanzas, Jonathan Mitchell, Tiago R. Simões, Fabien L. Condamine, Robin Aguilée, Pablo Peláez-Campomanes, Sabrina Renaud, Jonathan Rolland, Philip C. J. Donoghue

**Affiliations:** 1 Institut des Sciences de l’Évolution (ISE-M, UMR 5554, CNRS/UM/IRD/EPHE), Université de Montpellier, 34090 Montpellier, France; 2 Departamento de Paleobiología, Museo Nacional de Ciencias Naturales-CSIC, 28006 Madrid, Spain; 3 Department of Biology, West Virginia University Institute of Technology, 410 Neville Street, Beckley, WV 25801, USA; 4 Museum of Comparative Zoology & Department of Organismic and Evolutionary Biology, Harvard University, Cambridge, MA 02138, USA; 5 Laboratoire Évolution & Diversité Biologique, Université Paul Sabatier Toulouse III, UMR 5174, CNRS/IRD, 31077 Toulouse, France; 6 Laboratoire de Biométrie et Biologie Evolutive, UMR 5558, CNRS, Université Claude Bernard Lyon 1, 69622 Villeurbanne, France; 7 School of Earth Sciences, University of Bristol, Bristol BS8 1RJ, UK

**Keywords:** taxonomy, morphometrics, phylogeny, evolution, morphological clock, molecular clock, biodiversity, palaeobiogeography, macroevolution

## Abstract

**Simple Summary:**

All life is derived from a single common ancestor, whose descendants coevolved with the planet, shaping the structure of biodiversity and the physical processes that operate on Earth. This complex history cannot be inferred solely by studying the genomes of living organisms, nor through analysis of the fossil remains of their extinct relatives. Only a unified approach integrating living and extinct species and drawing from both genomic and anatomical evidence can achieve this aim. In this review, we highlight recent advances, challenges, and opportunities in this endeavour. These include the development of models for analysis of anatomical data; methods for combined analysis of fossil and living species, as well as anatomical and genomic data; and the combined estimation of evolutionary relationships, geographic range, and evolutionary rates. However, the application of such methods is limited by a shortage of expertise in taxonomy and comparative anatomy, which are skills required for the compilation of anatomical datasets. Whereas there is a common concern for the incompleteness of the fossil record, knowledge with respect to the comparative anatomy of living species is equally incomplete. We anticipate that the increased demand for an integrative phylogenetic approach to reconstruct the tree of life and evolutionary patterns and processes will encourage researchers to overcome these challenges with the aim of elucidating the complexities behind organismal evolution across broad taxonomic and time scales.

**Abstract:**

The modern era of analytical and quantitative palaeobiology has only just begun, integrating methods such as morphological and molecular phylogenetics and divergence time estimation, as well as phenotypic and molecular rates of evolution. Calibrating the tree of life to geological time is at the nexus of many disparate disciplines, from palaeontology to molecular systematics and from geochronology to comparative genomics. Creating an evolutionary time scale of the major events that shaped biodiversity is key to all of these fields and draws from each of them. Different methodological approaches and data employed in various disciplines have traditionally made collaborative research efforts difficult among these disciplines. However, the development of new methods is bridging the historical gap between fields, providing a holistic perspective on organismal evolutionary history, integrating all of the available evidence from living and fossil species. Because phylogenies with only extant taxa do not contain enough information to either calibrate the tree of life or fully infer macroevolutionary dynamics, phylogenies should preferably include both extant and extinct taxa, which can only be achieved through the inclusion of phenotypic data. This integrative phylogenetic approach provides ample and novel opportunities for evolutionary biologists to benefit from palaeontological data to help establish an evolutionary time scale and to test core macroevolutionary hypotheses about the drivers of biological diversification across various dimensions of organisms.

## 1. Introduction

Establishing an evolutionary time scale is a fundamental yet elusive goal of the Earth and life sciences. Without knowledge of the timing of evolutionary events, it is not possible to test hypotheses of ecological and evolutionary processes over geologic time. The fossil record once constituted the gold standard with respect to attempts to establish evolutionary time scales; however, for more than 50 years [1], that role has been filled by molecular clock approaches for groups with extant representatives. The benefits of analysing and integrating multiple lines of evidence to test hypotheses in science were previously tackled by Kluge [2] in what he called “TOTAL EVIDENCE analysis”. This idea was expanded by Nixon and Carpenter [3] in their “simultaneous analysis”. Since then, multiple Bayesian methods have been developed to accommodate genomic and/or morphological data.

A molecular clock methodology has also been developed to account for variation in the rate of molecular evolution among lineages and to accommodate the inaccuracies and imprecision inherent in the use of fossil evidence with respect to calibration [4,5,6], and it is now generally considered to be the most efficient methodology for calibrating evolutionary trees to geologic time. Therefore, evolutionary trees are often built on genomic datasets, putting morphology to one side [7]. However, fossil data provide the key means of clock calibration and are fundamental to the molecular clock methodology [5,8].

Traditionally, molecular clocks use fossil taxa to calibrate the divergences between living lineages (node dating). Nevertheless, the latest methods (tip dating) allow fossil species to be included alongside their living relatives, with the absence of molecular sequence data for fossil taxa remedied by supplementing the sequence alignments for living taxa with phenotype character matrices for both living and fossil taxa in total evidence dating [9,10]. In this way, the temporal constraints on lineage divergence provided by fossil species can be implemented in a more direct manner. Building total-evidence time-calibrated phylogenies is critical to increase the accuracy of the inferences regarding macroevolutionary processes. Tip dating is being increasingly applied with combined datasets, and it has begun to be used in fossils and/or living morphological datasets alone [11] in what has been called the morphological clock. Morphological data are a crucial component of phylogenetic inference, as they are usually the only information available to integrate both living and extinct members of an evolutionary tree [12]. The importance of morphological phylogenetics for dating the tree of life is widely recognized and has been bolstered by recent methodological developments. Statistical techniques, mostly using Bayesian inference, now allow researchers to test and implement variations in clock models, data partitioning, taxon sampling strategies, sampling of ancestors, and tree models (e.g., the fossilized birth–death (FBD) tree model) using morphological data [13,14,15,16,17,18,19]. In this way, palaeontologists are now able to achieve more accurate modelling of the diversification process across geological time, a crucial aspect of phylogenies with taxonomic sampling extending into deep time. Over the last years, the concurrent discovery of new fossil sites in previously rarely explored areas, the improvement of dating techniques, and the development of effective and integrative methods in phylogenetics have revitalized the study of speciation and extinction rates, as well as their variation over time and among clades [20,21,22]. Phylogenetic approaches in macroevolution enable diversification rates to be tied to changes in paleoenvironmental (extrinsic) and/or biotic (intrinsic) factors. These state-of-the-art approaches can be used to establish a time scale for evolution, linking phenotypic evolution with diversification rates and extrinsic phenomena, including causal agents of evolutionary change, such as global climate oscillations [23,24,25]. Given that phylogenies with extant taxa do not contain enough information for macroevolutionary dynamics to be fully and reliably reconstructed [26,27], phylogenies must include both extant and extinct taxa, which can only be achieved through integration of phenotypic data. In fact, a comprehensive understanding of evolution requires fossil data. Unfortunately, morphological phylogenetic data are lacking for most groups. Moreover, the morphological characteristics of living taxa are usually overlooked, and the data needed to determine the phylogenetic positions of fossil taxa with respect to their present-day relatives are often unavailable for many clades.

Given that establishing evolutionary time scales is a key goal of palaeontology, it is surprising that these phylogenetic methods are not more widely adopted by palaeontologists. Hence, the goal of this paper is to highlight some of the latest methodological advances bridging extinct and extant organismal biology that will help palaeontologists to address key aspects of patterns and processes in evolution.

## 2. Advances in Integrated Phylogenetics

### 2.1. Taxonomy

Taxonomy has been marginalized and traditionally treated as a purely descriptive discipline for both extant and extinct organisms [28]. However, the discovery of new fossils from key but underexplored areas of the world and/or key time intervals in the history of life are crucial to evolutionary biology. The study of new fossil taxa can shed light onto phylogenetic relationships in order to infer the time in which anatomical novelties appeared in a given group, as well as the biotic/abiotic factors driving their origin. Taxonomic studies in palaeontology are crucial for tackling all biochronological palaeobiogeographical and macroevolutionary questions. Discovery and description of new species creates generate raw data for further analysis by providing information on character states (and therefore phylogenetic inference), biogeographical locations, and temporal calibrations that are foundational to dating and reconstructing the evolutionary history of life. For instance, the study of the first Neogene micromammals from Zahlé (Bekaa Valley) discovered in Lebanon, one of the only two terrestrial Late Miocene sites in the Arabian Peninsula, has provided relevant data concerning new species situated at pivotal phylogenetic positions. This has allowed for inference of the expected dental morphology of the ancestors of some important lineages of rodents [29], as well as the evolutionary history of such important genera as *Progonomys*, the earliest known murine (Old World mice and rats), which is the first modern representative of the group to spread out of southern Asia [30]. Moreover, these data were relevant with respect to inferring the age of the sites (several million years older than previously thought), as well as the timing and nature of the migration events that took place between Eurasia and Africa via the Arabian plate.

### 2.2. Morphological Datasets

Modelling the evolution of morphological structures is a complex but crucial task for improving the practice of morphological phylogenetics and for testing evolutionary scenarios. The ability of morphological data to place extinct taxa phylogenetically is widely acknowledged, as sampling fossils for molecular data is typically impossible [31,32,33]. Fossil data are fundamental to molecular clocks, providing the key means of time calibration, although their commonplace use is far from satisfactory [5,8]. It is essential that the phylogenies of fossil species used in molecular clock calibration be compatible with the phylogenies of the living species that underpin the divergence time analysis. To this end, it is essential that taxonomists gather phenotypic information at the level of individual species, as their molecular counterparts do, instead of as usual, gathering such phenotypic information at a higher level (e.g., genus) [7]. Therefore, an important issue faced by taxonomists is the scarcity of morphological datasets of whole clades at the species level. Surprisingly, sourcing morphological datasets for living species may be more challenging than for their fossil counterparts [34]. Unfortunately, there is a continuously decreasing number of taxonomists able to collect and analyse phenotypic data [7], and even if the need for such expertise is pressing, taxonomical studies are still considered unfashionable instead of being encouraged.

Phylogenetic morphological datasets are frequently composed of discrete characters only or continuous characters discretized into arbitrary categories. However, discrete and continuous characters are jointly evolving, and the latter may contain information concerning gradual variations; ignoring this mutual information may lead to biased parameter estimates [35,36]. There is a long-standing debate in the scientific community concerning the use of quantitative characters for phylogenetic reconstructions, with disagreements concerning their suitability for inferring phylogenies [37,38]. However, continuous traits reduce the subjective bias of discrete characters and represent the full range of interspecific variation; therefore, they can be useful in phylogenetic reconstructions. Geometric and morphometric methods were applied as early as the 1990s to characterize fossil rodent taxa, to assess their relationship with relatives, and quantify evolutionary patterns [39]. Since then, the field has been renewed by the rise of 3D methods [40], enabled by the increasing availability of µCT scanners. A recent avenue of research with involves the joint use of geometric morphometrics and phylogenetic methods to map the evolution of complex structures and test models of evolution [41]. A particular challenge is constituted by the multivariate nature of morphometric data, although phylogenetic models are being developed to accommodate such issues [36,42,43]. It is now possible to use multivariate data directly in divergence time estimation [44].

Another challenge involves integrating developmental constraints on the evolution of morphological character, such as serial homologies and correlated characters, into phylogenetic models [45].

Although further work is needed to solve many of the concerns surrounding continuous data, new approaches are being developed [46,47,48] to analyse and understand the nature of these characters so that they can be used in support of ‘total evidence’ analyses.

Recent developments in morphometrics, phylogenetics, and comparative methods have revitalized the use of morphological data by palaeontologists to elucidate the dynamics of evolution over time [13,16,21,49,50,51,52,53]. Therefore, a new era of high-impact and interdisciplinary morphological taxonomy is beginning.

### 2.3. Calibrating the Tree of Life

Time provides palaeontologists with a unique perspective on phylogenetic history. Maximum parsimony was, until recently, the only way for palaeontologists to analyse morphological datasets. Despite initial attempts to integrate stratigraphic data with parsimony analyses in the 1990s [54,55], the problem faced by palaeontologists had was the considerable time and effort they had to dedicate to manually calibrate the resulting trees. Moreover, subject to the number of taxa included in a dataset, palaeontologists had to infer the distribution of morphological characters without including temporal data, with a consequent loss of information. The recent development of methodological approaches facilitating a posteriori time calibration of phylogenetic trees, such as PaleoTree [56,57] or STRAP (Stratigraphic Tree Analysis for Palaeontology) [58] allows for time calibration of phylogenetic trees resulting from parsimony analyses, as well as assessment of their agreement with the stratigraphic record (stratigraphic congruence) [58,59,60,61]. Owing to the development of such methods, the incorporation of stratigraphic data into parsimony analysis has been bolstered, presenting the opportunity to use additional techniques of phylogenetic reconstruction with morphological data [52,58,61,62].

**Figure 1:**
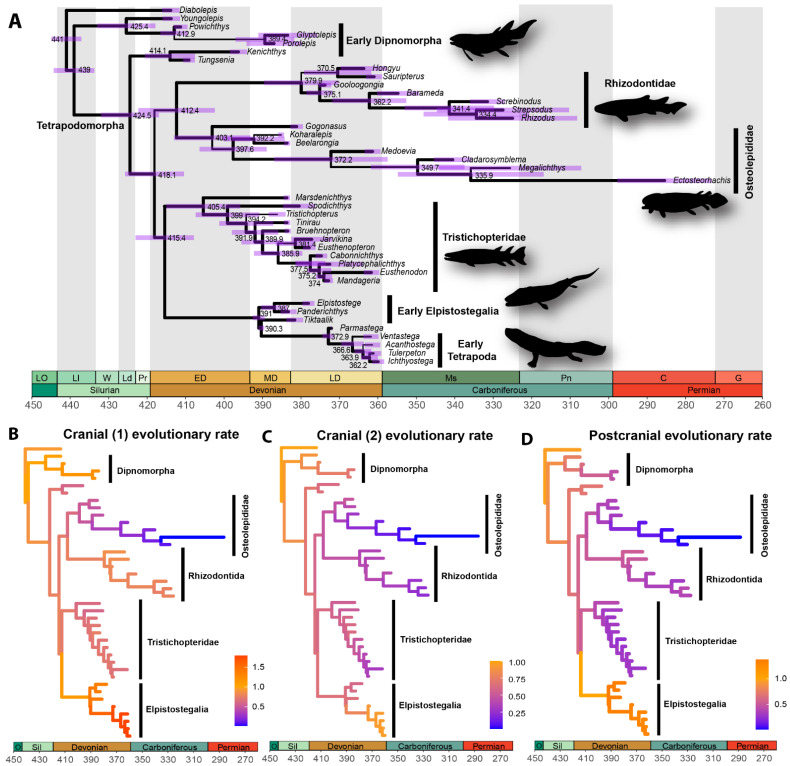
Bayesian evolutionary tree with estimated divergence times and evolutionary rates for the major groups of early tetrapodomorphs (adapted from [67]). (**A**) Divergence times for the fish–tetrapod transition. Node values represent median ages; purple error bars represent the 95% highest posterior density (HPD) intervals; branch thickness is proportional to posterior probabilities. (**B**–**D**) Relative rates of morphological evolution across subdivisions (partitions) of the phenotype in early tetrapodomorphs: two partitions including cranial characters and one partition including postcranial characters. All silhouettes created by TRS.

Since the introduction of Bayesian tip-dating phylogenetic methods, which were first applied with simplified clock and tree models [9,10], the inclusion of stratigraphic data into phylogenetic analyses has boomed [52,63,64]. The development of tip dating with more complex mechanistic tree models, such as the fossilized birth–death (FBD) tree [65,66] and its subsequent variations—such as the skyline FBD [13,14,15,16,17,18,19], which enables speciation, extinction, and fossilization parameters to be changed piecewise across the tree—allowed fossil species to be analysed in conjunction with and within the same analytical framework as extant taxa using Bayesian phylogenetics. This has been particularly useful for palaeontologists, who have revitalizing the use of morphological data to elucidate the dynamics of evolution across the tree of life [16,17,49,51,52,63,67,68,69] (Figure 1). These analyses can be carried out with morphological datasets alone, in what is called the morphological clock [16,17,51,52,63,70], which adds another method of reconstructing evolution to the palaeontologist’s toolbox. A morphological clock can be applied with data from extinct clades only [17,52,60,63,67,71] or with data from fossils and extant taxa [16,49,51,70,72,73]. Therefore, these recent developments applying Bayesian methods using fossil taxa as tips make it possible to compare phylogenies of extinct taxa obtained by means of evolutionary models with those resulting from maximum parsimony, which remains the most widely applied method for analysis of morphological data. The availability and inclusion of fossils in analysis enables Bayesian tip dating, which may improve the accuracy and precision of divergence time estimates [74,75]. Nevertheless, tip calibration has been shown to lead to ‘deep root attraction’ [76,77,78]. However, this artefact can be mitigated by using informative priors for FBD parameters [16,77] or a combination of tip and node calibration (whereby in the absence of tree-internal clade age constraints, the age estimates are unbounded by anything other than the root, leading to ages that become more precise with proximity to the root) [79].

Morphological data can be combined with a molecular matrix either based on a few loci (generated with Sanger techniques) [49] or based on hundreds of loci (generated with next-generation sequencing techniques) [80]. A morphological clock is then integrated, along with several molecular clocks, taking into account rate and clock heterogeneity across the dataset. To date, most total-evidence dating analyses have been carried out with molecular matrices composed of a few loci. However, the bird order Sphenisciformes (penguins), for instance, has been studied using both, a few [49] and hundreds of loci [80] and recovered similar results. Future studies will rely on vast ranges of genomic data combined with morphological datasets to estimate phylogenetic relationships and divergence times. A challenge is to infer to what extent the inclusion of increasingly large molecular datasets in combined analyses could affect to the phylogenetic contribution of morphological data. According to Neumann [81], they will continue to have a strong influence, even when outnumbered by molecular data by thousands of times.

Challenges remain for palaeontologists because for many important groups (e.g., rodents), the number of characters available for computational morphological phylogenies is very limited, and commonly, the relationships between taxa are inferred by hand rather than by computational algorithms. The main issue is that building new phylogenies from new character matrices of morphological data is very time-consuming. Systematics must be revitalized and encouraged more than ever. There is a need for palaeontologists and neontologists capable of encapsulating systematic data to infer testable systematic hypotheses [82].

### 2.4. Exploring Macroevolution

The combination of evidence from species-level phylogenies (with extinct and extant taxa) with robust estimates of divergence time is thus vital to infer biogeographical and macroevolutionary patterns within and among clades. [7,83]. Moreover, the integration of both morphological and molecular data for Bayesian relaxed clock analysis (total-evidence dating) provides a joint estimate of tree topology, divergence times, and evolutionary rates in a multivariate statistical framework [14,70,84]. Total-evidence dating has also been improved by the development of the FBD process to estimate more accurate priors on times [66,84]. Molecular data improve relationship information among living taxa and help to (re-) optimize the morphological characters, improving their ability to accurately place fossils [7].

**Figure 2:**
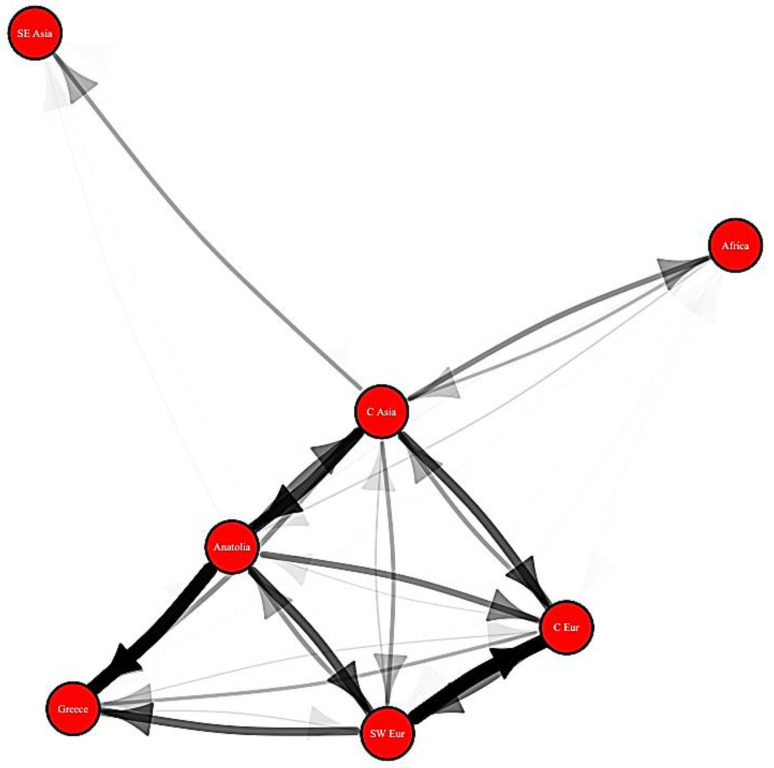
Map of reconstructed immigration and emigration rates for Old World Miocene muroids (work in prep.) based on a DEC analysis run using BioGeoBears in R. Arrows represent reconstructed movement of an individual lineage from one region to another. Arrows are shaded to represent the frequency of a specific transition.

Ancestral state estimations represent a central tool for the exploration of trait evolution. They are useful to test hypotheses, such as the biogeographic history and movements of clades through time (e.g., [85,86]), as well as the order and timing of character state changes (e.g., [87,88]). Species distributions are defined by presence or absence in pre-defined geographic units. The most likely biogeographical scenarios at all internal nodes of a given time-calibrated evolutionary tree can be estimated using maximum likelihood or Bayesian approaches, notably with the dispersal–extinction–cladogenesis (DEC) model [85,89,90,91,92]. The DEC model and its derivatives [93] allow for investigation of time-calibrated phylogenies with extant and extinct taxa while considering tectonic evolution via the incorporation of time bins in which the connectivity between any two areas can change through time [94,95]. Geological connectivity can be coded as a matrix of connection/disconnection relying on the latest palaeogeographical scenarios (e.g., [96]) for a given region. The DEC model can also incorporate trait-dependent models in which species traits can influence dispersal rates [97]. A DEC model was applied to a dataset of European fossil muroids to reconstruct numerous transitions, revealing the most often utilised migration corridors for these ancient rodents (Figure 2). This analysis exemplifies how the combination of phylogenetic models and fossil data can produce novel insights into the structure of ancient communities and their biogeographic habits.

Phylogenetic approaches in macroevolution now allow diversification rates be tied to changes in paleoenvironmental (extrinsic) and/or biotic (intrinsic) factors [98,99,100]. Relaxed clock Bayesian inference methods under the FBD tree model and its skyline variant, SFBD, allow speciation, extinction, and fossilisation parameters to vary across time bins [13,19]. Such model developments can provide more precise estimates of macroevolutionary parameters, including net diversification, turnover, and fossil sampling rates [17]. Some authors have called into question estimation of speciation and extinction rates from extant trees [26,101,102]. However, even if their limitations have been, in part, overcome by recent methodologies [103], phylogenies that combine palaeontological and neontological data have been proven to provide accurate insights into macroevolutionary dynamics [83,104,105]. This is especially evident in groups with high past diversity but which are currently extinct or only represented by a few taxa [105].

By inferring the diversification rates using several methods with different model assumptions and focusing on clades and lineages with rate shifts that are consistently estimated regardless of method, it is possible to reliably focus on the possible causes of those shifts [22]. Phylogenetically informed hierarchical Bayesian regression [106,107] is a general tool that has only recently begun to be explored. By allowing multiple factors to influence diversification rates and pooling the estimates across time and space, many parameters can be regularised to identify factors that have the largest effect on rate variation. This allows for testing of more nuanced hypotheses; instead of investigating whether abiotic or biotic factors were more important, the relative importance of many different factors can be simultaneously estimated, and their interactions can be investigated [108,109]. Using hierarchical regressions, it is possible to estimate clade-specific values that represent the unmeasured variables and assess how multiple distinct lineages differ in their evolutionary responses to climatic shifts during a given period of time and control for differing geographic locations. Using temporally, geographically, and phylogenetically well-resolved datasets to pool parameter estimates by region and clade allows for exploration of how climatic (Court Jester) and ecological (Red Queen) factors influenced diversification at a level of resolution that has not been achieved to date.

Smits [109] fitted Bayesian hierarchical models to the durations of brachiopod lineages over time and estimated the relative importance of factors such as geographic range, environmental preference, and body size on extinction intensity. This allowed the author to go beyond simply asking whether Court Jester or Red Queen effects predominated but instead to delve into how the relative importance of trait-based or environmentally based factors changed over time in these lineages. Smits was able to use the difference between overall fitness (total duration) and the strength of selection (trait-specific regression coefficients of duration) to show how background and mass extinctions vary in their selective regimes.

Shifts of diversification across a phylogeny can provide lines of evidence for the respective role of biotic and climatic variables in macroevolution [22,110]. A major outstanding question in macroevolution is the extent to which diversification rates are influenced by organismal traits and environmental changes. To differentiate between the Court Jester (extrinsic controls, such as environment and geography) and Red Queen (intrinsic controls, such as traits) hypotheses in shaping the radiation of a given group, palaeontologists have to provide a wealth of data from large geographical areas over long temporal intervals. Smits [108] used a hierarchical Bayesian model to determine which parameters best explain the durations of North American fossil mammal species over the Cenozoic. This flexible approach facilitated estimation of how different factors, ranging from geographic region to locomotor, and dietary categories influenced extinction risk in Cenozoic mammals while accounting for unobserved clade- and species-specific factors.

Repeated convergent trait evolution in clades allows for an examination of the role disparity plays while minimising the effects of phylogenetic pseudoreplication [111,112]. Probabilistic models, such as fossil BAMM [104] or PyRate [113], have been developed to estimate the rates of diversification and preservation using time-calibrated trees and fossil occurrences. Probabilistic models incorporate the distribution of lineage durations (along with the number of fossil occurrences to estimate a preservation rate) to estimate the optimal combination of speciation and extinction events explaining the shape and distribution of branch lengths in a phylogeny.

Uncertainty exists with respect the measured traits, the shape of the phylogeny, and the estimates of the rates themselves. Fuentes-G. [114] recently extended phylogenetic regressions to accommodate these different levels and degrees of uncertainty. In their study [114], they explored how allometric relationships vary in posture across a phylogeny of mammals, although their flexible approach is applicable to any dataset.

Such models are increasingly used in palaeobiological and macroevolutionary studies [108,109,114,115,116,117,118]. Once rates have been estimated using these approaches, hierarchical Bayesian regressions [108] can be used to identify associations between diversification rates and abiotic/biotic variables (such as climate, traits, and local richness) to evaluate the relative importance of each variable in driving diversification. Cole [115] examined the effect of the importance of environmental and habitat-based traits relative to traits focused on feeding mechanisms and selectivity on the extinction propensity of crinoids. This study demonstrated that the same biotic trait (body size) could have opposite effects on extinction depending on the abiotic environment (mixed or siliciclastic) in these crinoids. The ability to test nuanced hypotheses such as that outlined above, whereby environmental conditions influence not only the diversification rates themselves but also the importance and directionality of different biotic traits (and potentially vice-versa) is a recent and exciting development.

## 3. Conclusions

The statistical techniques mentioned above have only begun to be applied to questions in palaeontology over the past decade but have found extensive applications in phylogenetic comparative analysis, quantitative genetics, and ecology. Complementary methodologies that combine morphological and molecular approaches can provide novel answers to broad evolutionary and deep-time questions with methods to infer the dynamics of speciation and extinction, as well as the variation in species diversification among lineages, using time-calibrated phylogenetic trees. These recent developments provide palaeontologists with a golden opportunity to considerably expand their research toolkit and bridge emerging techniques from evolutionary biology and paleobiology. In paleontological research the many challenges with respect to our understanding of how life has evolved and survived on Earth have to be approached in a collaborative and integrative fashion. Many of the most important problems can now be solved with interdisciplinary teams of scientists using the best available technology. However, it is crucial that all scientist inside and outside the discipline restore the place of palaeontology at the high table of evolutionary biology where it belongs.

## Data Availability

Not applicable.
